# Exercise as cancer treatment: A clinical oncology framework for exercise oncology research

**DOI:** 10.3389/fonc.2022.957135

**Published:** 2022-09-02

**Authors:** Kerry S. Courneya, Christopher M. Booth

**Affiliations:** ^1^ Faculty of Kinesiology, Sport, and Recreation, College of Health Sciences, University of Alberta, Edmonton, AB, Canada; ^2^ Department of Oncology, Queen’s University, Kingston, ON, Canada; ^3^ Cancer Care and Epidemiology, Cancer Research Institute, Queen’s University, Kingston, ON, Canada

**Keywords:** exercise, physical activity, cancer therapy, tumor growth, survival

## Abstract

Exercise has been proposed as a possible cancer treatment; however, there are an infinite number of clinical oncology settings involving diverse cancer types and treatment protocols in which exercise could be tested as a cancer treatment. The primary purpose of this paper is to propose a conceptual framework to organize and guide research on exercise as a cancer treatment across distinct clinical oncology settings. A secondary purpose is to provide an overview of existing exercise research using the proposed framework. The Exercise as Cancer Treatment (EXACT) framework proposes nine distinct clinical oncology scenarios based on tumor/disease status and treatment status at the time of the proposed exercise treatment. In terms of tumor/disease status, the primary tumor has either been surgically removed (primary goal to treat micrometastases), not surgically removed (primary goal to treat the primary tumor), or metastatic disease is present (primary goal to treat metastatic disease). In terms of treatment status, the extant disease has either not been treated yet (treatment naïve), is currently being treated (active treatment), or has previously been treated. These two key clinical oncology variables—tumor/disease status and treatment status—result in nine distinct clinical oncology scenarios in which exercise could be tested as a new cancer treatment: (a) treatment naïve micrometastases, (b) actively treated micrometastases, (c) previously treated micrometastases, (d) treatment naïve primary tumors, (e) actively treated primary tumors, (f) previously treated primary tumors, (g) treatment naïve metastatic disease, (h) actively treated metastatic disease, and (i) previously treated metastatic disease. To date, most preclinical animal studies have examined the effects of exercise on treatment naïve and actively treated primary tumors. Conversely, most observational human studies have examined the associations between exercise and cancer recurrence/survival in patients actively treated or previously treated for micrometastases. Few clinical trials have been conducted in any of these scenarios. For exercise to be integrated into clinical oncology practice as a cancer treatment, it will need to demonstrate benefit in a specific clinical setting. The EXACT framework provides a simple taxonomy for systematically evaluating exercise as a potential cancer treatment across a diverse range of cancer types and treatment protocols.

## Introduction

Exercise has demonstrated many supportive care benefits for cancer patients; however, it has also been proposed as a possible treatment for cancer based on emerging preclinical, observational, and clinical research (1, 2). In animal models, exercise is typically evaluated for its ability to directly affect the growth and spread of an untreated primary tumor (3) or to potentiate the effects of an existing cancer treatment on a primary tumor (4). In human research, exercise is most often evaluated for its ability to reduce the risk of recurrence and improve survival in postsurgical early stage cancer patients without regard to treatment status (5). The clinical scenario in which exercise might slow the growth and spread of an existing untreated primary tumor is very different than the one in which exercise might lower the risk of cancer recurrence or death in postsurgical early-stage cancer patients during or after adjuvant therapy.

As noted by Ashcraft et al. (3), exercise researchers conducting preclinical animal studies should design studies that reflect current clinical oncology practice. This same guidance is also important for exercise researchers conducting human studies. Unfortunately, there are an infinite number of clinical oncology settings involving diverse cancer types and treatment protocols in which exercise could be tested as a cancer treatment. A conceptual framework to organize and characterize the critical aspects of a clinical oncology setting may allow for a more systematic approach to the study of exercise as a cancer treatment across a wide range of cancers and treatment protocols. The primary purpose of the present paper is to propose a conceptual framework for identifying distinct clinical oncology scenarios in which exercise may be tested as a cancer treatment. A secondary purpose is to provide an overview of existing animal and human exercise research and offer directions for future research across these distinct clinical scenarios.

## Exercise as cancer treatment

The potential roles of exercise as a cancer treatment are informed by its unique characteristics compared to existing cancer treatments. Physical activity is defined as any bodily movement produced by contraction of the skeletal muscles that results in a substantial increase in energy expenditure over resting levels. Exercise is a subset of physical activity consisting of planned, structured, and repetitive bodily movement performed to improve or maintain components of physical fitness such as cardiorespiratory endurance, muscular strength, muscular endurance, body composition, flexibility, and balance. Consequently, unlike existing cancer treatments, exercise is a behavior that can only be self-administered by the patient. Moreover, unlike most existing treatments, exercise offers numerous other health benefits and few adverse effects which establish a favorable benefit-to-harm ratio even in the absence of a survival benefit.

Additionally, although a single administration of an exercise session can have acute biological effects, it will not eliminate an existing tumor like a surgical resection. In this sense, exercise is more similar to radiation therapy or drug therapy in that it will need to be delivered in numerous “fractions” or “cycles” over an extended period of time to achieve a clinical benefit. Furthermore, exercise is essentially a single drug (energy expenditure) whose biological effects can be manipulated based on the type, dose, and administration schedule (i.e., the exercise prescription). Similar to other drugs, exercise has systemic effects, allowing it to potentially act as a treatment for local, regional, and distant disease. Also similar to other drugs, exercise can be administered at a maximally tolerated dose (e.g., high intensity, high volume, combined modality) for a short period of time (similar to an adjuvant therapy) or it can be administered at a more tolerable dose (e.g., walking, light-moderate intensity exercise) for an extended period of time (similar to a maintenance therapy). Based on these characteristics, exercise as a cancer treatment is best conceptualized as a single agent drug (energy expenditure) with many possible administration schedules that may or may not have anti-cancer properties. Importantly, like other cancer treatments (6), exercise also imposes a substantial time toxicity that must be balanced against any demonstrated benefit (**Table 1**).

**Table 1:** Estimated patient time commitment for exercise treatment compared to standard protocols for other cancer treatment modalities.

Treatment	Frequency	Administration Time^1^	# of Administrations	Standard Protocol	Total Time
		(per single session)	(per standard protocol)	Length/Duration	Commitment^2^
Exercise therapy					
Shorter duration^3^	3-7 days/week	30-60 minutes	12-182	4-26 weeks	6-182 hours
Longer duration^4^	3-7 days/week	30-60 minutes	156-365/year	Years^5^	78-365 hours/year
Surgery	Once	Several hours	1	Days/weeks	Days/weeks
		(plus recovery)			
Radiation therapy	5 days/week	15 minutes	25-30	5-6 weeks	6-8 hours
(external beam)					
Chemotherapy	Every 2-4 weeks	Several hours	4-6	4-6 months	12-18 hours
(infusion)					
Immunotherapy	Every 2-4 weeks	10 minutes	12-26	1-2 years	2-8 hours
(injection)					
Hormone therapy	Daily	1 minute	1,825-3,650	5-10 years	30-60 hours
(oral)					

^1^Does not include travel time, waiting time, or observation/recovery time after treatment.

^2^Does not include time commitments beyond treatments such as lab work, imaging, biopsies, and managing adverse events. ^3^Shorter duration protocols may apply to clinical settings such as pending primary treatment, pending adjuvant treatment, or during active treatment. ^4^Longer duration protocols may apply to clinical settings such as postsurgical surveillance, active surveillance, or posttherapy surveillance. ^5^Unclear how many years of exercise would be necessary for a clinical benefit.

## The exercise as cancer treatment framework

**Figure 1:**
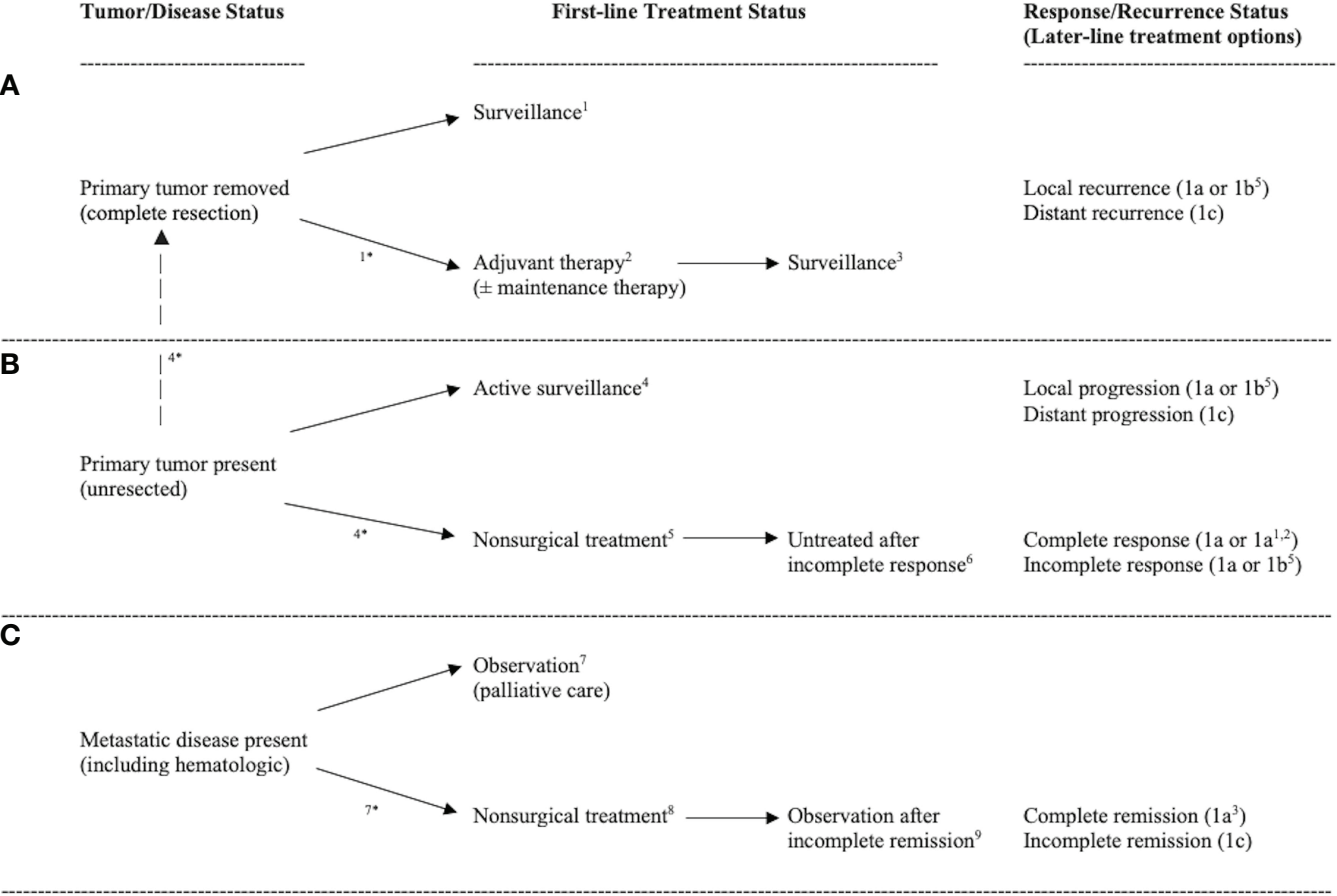
Diagram of common clinical oncology settings in which the primary tumor is removed **(A)**, the primary tumor is present **(B)**, or metastatic disease is present **(C)** leading to nine distinct clinical oncology scenarios. Note: ^1^treatment naïve micrometastases, ^2^actively treated micrometastases, ^3^previously treated micrometastases, ^4^treatment naïve primary tumors, ^5^actively treated primary tumors, ^6^previously treated primary tumors, ^7^treatment naïve metastatic disease, ^8^actively treated metastatic disease, ^9^previously treated metastatic disease. ^*^clinical oncology scenario occurs because of pending treatment. Response/recurrence status indicates possible disease/treatment outcomes leading to repetition of the clinical oncology settings (i.e., second-line and later-line therapies).

For exercise to be integrated into clinical practice as a cancer treatment, researchers will need to demonstrate benefit in a specific clinical setting involving a specific cancer type and treatment protocol. In clinical oncology, however, there are an infinite number of unique settings in which exercise could act as a cancer treatment. The Exercise as Cancer Treatment (EXACT) framework proposes nine distinct clinical oncology scenarios based on two key clinical oncology variables at the time of the proposed exercise treatment–tumor/disease status and treatment status (**Figure 1** **;** **Table 2**).

**Table 2:** Clinical targets of exercise as a cancer treatment based on tumor/disease status and treatment status.

	Treatment Status		
Tumor/disease Status	Treatment naïve	Active treatment	Previous treatment
Primary tumor removed	Direct effects on treatmentnaive micrometastases(prevent recurrence/metastases)	Direct and treatment interaction effectson actively treated micrometastases(prevent recurrence/metastases)	Direct effects on previouslytreated micrometastases(prevent recurrence/metastases)
			
Primary tumor present (de novo or recurrent)	Direct effects on treatmentnaive primary tumor(slow/prevent primary tumor growth and spread)	Direct and treatment interaction effectson actively treated primary tumor(reduce/eliminate primary tumor growth and spread)	Direct effects on previouslytreated primary tumor(slow/prevent primary tumor growth and spread)
			
Metastatic disease present(de novo or recurrent)	Direct effects on treatmentnaïve metastatic disease(slow/prevent metastaticdisease growth and spread)	Direct and treatment interaction effectson actively treated metastatic disease(slow/reduce metastatic diseasegrowth and spread)	Direct effects on previouslytreated metastatic disease(slow/prevent metastatic diseasegrowth and spread)

In terms of tumor/disease status, at the time of the proposed exercise treatment the primary tumor has either been surgically removed (**Figure 1A**), not surgically removed (**Figure 1B**), or metastatic disease is present (**Figure 1C**). If the primary tumor has been surgically removed, the main goal of exercise is to eliminate any cancer cells that may have escaped the primary tumor (herein referred to as micrometastases). If the primary tumor has not been completely resected, the main goal of exercise is to treat the primary tumor (and any potential micrometastases). If metastatic disease is present (including hematologic cancers), the main goal of exercise is to treat the disseminated disease. Tumor/disease status is important because treating the primary tumor, micrometastases, or metastatic disease is distinct given the genetic and epigenetic differences between metastases and primary tumors (7, 8).

In terms of treatment status, at the time of the proposed exercise treatment the extant disease (primary tumor, micrometastases, and/or metastases) has either not been treated yet (treatment naïve), is currently being treated (active treatment), or has already been treated (previously treated). It is also possible that actively treated disease has been previously treated (i.e., second-line or later therapies) and that previously treated disease has been treated multiple times (i.e., heavily pretreated patients). Treatment status is important because existing and previous cancer treatments may alter the biology, genetics, and/or location of any remaining cancer (9) and, therefore, the effects of exercise may be different for treatment naïve, actively treated, and previously treated cancers.

These two key clinical oncology variables—tumor/disease status and treatment status—result in nine distinct clinical oncology scenarios in which exercise could be tested as a new cancer treatment across a wide range of cancer types and treatment protocols: (a) treatment naïve micrometastases, (b) actively treated micrometastases, (c) previously treated micrometastases, (d) treatment naïve primary tumors, (e) actively treated primary tumors, (f) previously treated primary tumors, (g) treatment naïve metastatic disease, (h) actively treated metastatic disease, and (i) previously treated metastatic disease. Depending on the response/recurrence status after first-line treatments, these scenarios may repeat themselves during second-line and later-line therapies (**Figure 1**). In the following sections, these distinct clinical oncology scenarios are described in more detail concerning their real-world clinical settings (**Table 3**), clinical goals and challenges, the treatment goals of exercise, the feasibility and likelihood of exercise producing a benefit (**Table 4**), existing animal and human exercise research, and future research directions (**Table 5**).

**Table 3:** Common clinical oncology settings based on tumor/disease status and treatment status in which exercise may be tested as a cancer treatment.

	Treatment Status		
Tumor/disease Status	Treatment naïve	Active treatment	Previous treatment

Primary tumor removed	Surgery onlyPending adjuvant therapy	Any adjuvant therapy setting	Any post adjuvant therapy setting
			
Primary tumor present (*de novo* or recurrent)	Pending primary treatmentActive surveillance	Any nonsurgical primary/neoadjuvanttherapy setting (or treatment of local recurrence)	Any post primary/neoadjuvant therapy setting without a complete response (or any early stage setting with a local recurrence)
			
Metastatic disease present (*de novo* or recurrent)	Treatment pending/unavailable	Any nonsurgical treatment setting	Any post metastatic treatment setting without a complete remission (or any early stage setting with a distant recurrence)

**Table 4:** Summary of key characteristics of the distinct clinical oncology scenarios in which exercise may be tested as a cancer treatment.

Distinct clinical oncology scenarios	Common clinical oncology settings	Clinical concern	Primary goal of exercise treatment	Exercise feasibility	Potential for exercise benefit
Treatment naïve micrometastases	Complete surgical resection of primary tumor followed by surveillance or pending adjuvant therapy	Possible micrometastases	Adjuvant therapy or maintenance therapy to eliminate treatment naive micrometastases	High, given complete resection and no adjuvant therapy although long term adherence may be a challenge	Low, given low likelihood of recurrence
Actively treated micrometastases	Complete surgical resection of primary tumor followed by adjuvant therapy	Known/suspected micrometastases	Concurrent adjuvant therapy to help eliminate actively treated micrometastases	Medium, given complete resection and short adjuvant therapy window but possible treatment side effects	Medium, given higher likelihood of recurrence and possible interactions with existing treatments
Previously treated micrometastases	Complete surgical resection of primary tumor plus previous adjuvant therapy followed by surveillance	Possible remaining micrometastases	Sequential adjuvant therapy or “switch” maintenance therapy to eliminate previously treated micrometastases	High, given complete resection and post adjuvant therapy recovery although long term adherence may be a challenge	Medium, given higher likelihood of recurrence offset by requirement to treat previously treated micrometastases
Treatment naïve primary tumors	Primary tumor pending treatment or immediate treatment is considered unnecessary	Growth and spread of primary tumor	Primary or induction therapy to reduce growth and spread of treatment naive primary tumor	High, given no treatments although long term adherence may be a challenge	High, given high likelihood of cancer progression and extended time frame for effects
Actively treated primary tumors	Primary tumor (and possible micrometastases) actively treated with nonsurgical primary or neoadjuvant therapy	Poor or incomplete response of primary tumor (and possible micrometastases) to therapy	Concurrent primary or neoadjuvant therapy to improve response of actively treated primary tumor (and possible micrometastases)	Medium, given short therapy window but possible treatment side effects	High, given modest rate of complete response and potential interaction with existing treatments
Previously treated primary tumors	Primary tumor (and possible micrometastases) previously treated with nonsurgical primary or neoadjuvant therapy without a complete response and with no immediate further treatment	Regrowth and spread of primary tumor (and possible micrometastases)	Sequential therapy or salvage therapy to slow regrowth and spread of previously treated primary tumor (and possible micrometastases)	Low, given likely short window between incomplete response and subsequent treatment	Low, given incomplete response of primary tumor and short window before additional treatment
Treatment naïve metastatic disease	*De novo* metastatic disease where no treatment options are available (or, more rarely, where initial management is observation)	Rapid progression of untreated metastatic disease	Induction therapy to slow growth of treatment naive metastatic disease	Low, given high symptom burden and limited life expectancy	Low, given untreated progressive disease and likely low exercise tolerance
Actively treated metastatic disease	*De novo* or recurrent metastatic disease actively treated with nonsurgical treatments	Poor response of metastatic disease to therapy	Concurrent therapy to improve response of actively treated metastatic disease	Low, given high symptom burden, treatment side effects, and limited life expectancy	Medium, given potentially responsive disease and potential interaction with existing treatments
Previously treated metastatic disease	*De novo* or recurrent metastatic disease previously treated with nonsurgical treatments without a complete remission	Rapid regrowth of metastatic disease	Salvage therapy to slow regrowth of previously treated metastatic disease	Low, given high symptom burden, treatment side effects, and limited life expectancy	Low, given untreated progressive disease and likely low exercise tolerance

**Table 5:** Summary of research evidence on exercise as a cancer treatment within the distinct clinical oncology scenarios.

Distinct clinical oncology scenarios	Animal studies (3)	Observational studies (5)	Clinical trials (10)	Overall evidence/future studies
Treatment naïve micrometastases	Few animal studies focused exclusively on this scenario.	No observational studies focused exclusively on this scenario. Some studies may have mixed this scenario with “actively treated micrometastases” and/or “previously treated micrometastases” scenarios.	No clinical trials on this scenario.	Very limited research on this scenario. Animal research suggests exercise may disrupt metastatic cascade. Animal studies and observational studies likely most feasible. Trials with circulating tumor markers may be feasible.
Actively treated micrometastases	Few animal studies focused exclusively on this scenario.	Few observational studies focused exclusively on this scenario. Many studies mix this scenario with “previously treated micrometastases” scenario. General finding is that higher postdiagnosis exercise is associated with lower risk of recurrence/death for some cancers.	Few clinical trials on this scenario. START trial (11) is one example.	Limited but promising research on this scenario. Need animal studies on this scenario and observational studies focused exclusively on this scenario. Clinical trials are feasible.
Previously treated micrometastases	No animal studies on this scenario.	Some observational studies focused exclusively on this scenario. Many studies mix this scenario with “actively treated micrometastases” scenario. General finding is that higher postdiagnosis exercise is associated with lower risk of recurrence/death for some cancers.	Few clinical trials on this scenario. CHALLENGE trial (12) is one ongoing example.	Limited but promising research on this scenario from observational studies. Need animal studies on this scenario and observational studies focused exclusively on this scenario. One phase III trial is ongoing.
Treatment naïve primary tumors	Most animal studies on this scenario. Most show that exercise slows the growth and spread of untreated primary tumors.	Very few observational studies on this scenario.	Few clinical trials on this scenario. ERASE trial (13) is one example using *in vitro* cell line. Australian trial (14) is one ongoing example.	Animal research very promising. Observational studies and clinical trials are needed. One phase II trial is ongoing.
Actively treated primary tumors	Some animal studies on this scenario. Most studies show that exercise improves chemotherapy efficacy.	Very few observational studies on this scenario.	Few clinical trials on this scenario. EXERT trial (15) is one example.	Animal research and phase I/II clinical trials are promising. Observational studies may not be critical as clinical trials are feasible. Larger clinical trials are needed.
Previously treated primary tumors	No animal studies on this scenario.	No observational studies on this scenario.	No clinical trials on this scenario.	Animal studies needed to determine if exercise can treat previously treated primary tumors. Clinical relevance of this scenario unclear.
Treatment naïve metastatic disease	Some animal studies on this scenario although most mix the occurrence and growth of metastatic tumors. Study findings mixed.	No observational studies focused exclusively on this scenario. Some studies mix this scenario with “actively treated metastatic disease” and/or “previously treated metastatic disease” scenarios.	No clinical trials on this scenario.	Animal research suggests an exercise effect but clinical benefit uncertain. Observational studies focused exclusively on this scenario are needed. Clinical trials may be challenging.
Actively treated metastatic disease	No animal studies on this scenario.	Few observational studies on this scenario. Most studies mix this scenario with “previously treated metastatic disease” scenario.	Few clinical trials on this scenario. HELP (16) is one example. Some trials mix this scenario with “previously treated metastatic disease”. Lung cancer trial (17) is one example and INTERVAL-GAP4 trial (18) is ongoing example.	Limited observational studies and clinical trials but promising. Animal studies needed. One phase III trial combining this scenario with “previously treated metastatic disease” scenario is ongoing.
Previously treated metastatic disease	No animal studies on this scenario.	Few observational studies on this scenario. Most studies mix this scenario with “actively treated metastatic disease” scenario.	No clinical trials exclusively on this scenario. Some trials mix this scenario with “actively treated metastatic disease”. Lung cancer trial (17) is one example and INTERVAL-GAP4 trial (18) is ongoing example.	Limited observational studies but promising. Animal studies needed. One phase III trial combining this scenario with “actively treated metastatic disease” scenario is ongoing.

## Scenario #1: Treatment naive micrometastases

The clinical scenario of “treatment naïve micrometastases” typically occurs after the complete surgical resection of an early-stage primary tumor where adjuvant therapy is either pending or not offered because the probability of micrometastases is either low or there is no established benefit of adjuvant therapy. In this scenario, any existing micrometastases are treatment naïve and, therefore, exercise is being proposed as a single modality adjuvant therapy or maintenance therapy (i.e., “adjuvant exercise therapy” or “maintenance exercise therapy). There are many real-world clinical settings where patients wait weeks for adjuvant therapy after surgery or where no adjuvant therapy is offered such as early-stage breast, prostate, colon, and bladder cancers. The clinical concern in this scenario, if any, is the possibility that some cancer cells (i.e., micrometastases) may have escaped the primary tumor and disseminated to other organs *via* the circulatory or lymph systems. The goal of exercise as a cancer treatment in this scenario, therefore, would be to eliminate any treatment naive micrometastases before they establish overt recurrent disease. Exercise as a monotherapy may act as a treatment for micrometastases through various mechanisms including increased fluid shear stress, enhanced immune surveillance, reduced inflammation, and improved insulin sensitivity (19). 

Given the relatively short window between surgery and planned adjuvant therapy, the feasibility of exercise in patients awaiting adjuvant therapy is limited and the prospect for an exercise benefit seems low. Conversely, the feasibility of an exercise intervention in patients who are not offered adjuvant therapy is high given that patients have only had surgery for a small tumor and are not receiving any adjuvant therapy. In this scenario, exercise may be tested as a high intensity, short term adjuvant therapy (i.e., months) or a lighter intensity, long term maintenance therapy (i.e., years), both of which may be quite feasible. Moreover, even though there is substantial time for exercise to affect outcomes in patients not offered adjuvant therapy (years or decades), the prospect for a meaningful cancer benefit will be related to the specific cancer type and the risk of disease recurrence. In exercise-sensitive cancers and high-risk clinical scenarios where relapse rates are substantial, exercise may offer larger benefits.

To date, there is limited animal and human exercise research that has examined exercise as a cancer treatment in this clinical scenario. A comprehensive systematic review of animal studies (3) identified only 10 of 53 studies that examined the effects of exercise using a metastasis model. In all studies, the (micro)metastases were treatment naïve. Moreover, only three studies utilized models in which metastases arose from a primary tumor with two reporting exercise had non-significant inhibition of tumor growth and the other reported accelerated tumor growth. Eight studies utilized intravenously injected tumor cells to establish metastases and reported mixed results in terms of lung tumor cell retention and the number of metastases. Some of these studies report both the emergence and growth of metastases, which reflect two distinct clinical oncology scenarios. Clearly, more preclinical research is needed that is relevant to this clinical scenario. To examine the effects of exercise on treatment naïve micrometastases in animal models, the most clinically relevant model would be to establish a primary tumor, allow it to disseminate, surgically remove it, and then randomize the animals to exercise or no exercise before any tumors develop (3, 8). The most clinically relevant outcome would be the recurrence of overt disease.

In human studies, clinical trials may be difficult to conduct in this scenario given the long-time frame and low likelihood of clinical events. Nevertheless, it may be possible to assess circulating biomarkers such as circulating tumor cells or circulating tumor DNA to select patients at higher risk of recurrence or to use as surrogate endpoints (20, 21). In this clinical scenario, observational studies may be most feasible and informative, despite their well-known limitations. To date, however, there is limited research using observational studies to examine the association between exercise and cancer outcomes in postsurgical cancer patients who have not received any adjuvant therapies (5).

## Scenario #2: Actively treated micrometastases

The clinical scenario of “actively treated micrometastases” typically occurs with the complete surgical resection of an early-stage primary tumor followed by a regional or systemic adjuvant therapy to treat known or suspected micrometastases (e.g., radiotherapy, chemotherapy, immunotherapy, hormone therapy). In this scenario where micrometastases are actively being treated, exercise is being proposed as a concurrent adjuvant or maintenance therapy (e.g., “adjuvant chemo-exercise therapy”, “adjuvant radio-exercise therapy”). There are many real-world clinical settings where this scenario occurs such as adjuvant chemotherapy for early-stage breast and colon cancers. The clinical concern in these settings is that the adjuvant therapy may not eliminate all the remaining cancer cells. The goal of exercise as a cancer treatment in these settings, therefore, would be to directly eliminate any remaining cancer cells (i.e., an additive effect) or to help existing adjuvant therapies eliminate any remaining cancer cells (i.e., interactive or synergistic effect). As noted earlier, exercise may directly treat micrometastases through various mechanisms, however, it may also potentiate the effects of existing treatments through similar mechanisms (e.g., increased fluid shear stress may make cancer cells more vulnerable to chemotherapy, or increased immune surveillance may enhance the effectiveness of immunotherapies). The feasibility of exercise in these clinical settings is more challenging because of possible treatment side effects, however, the exercise treatment only needs to be delivered during the existing adjuvant therapy (e.g., weeks to months). The potential benefit of exercise in these clinical settings will depend on the specific cancer type and treatment protocol, however, it may be higher given the higher rate of expected recurrence as well as the added potential of exercise to act as a “treatment sensitizer”.

To date, there are very few preclinical studies focused on exercise as a cancer treatment in this scenario. To examine the effects of exercise on actively treated micrometastases in animal models, the most clinically relevant model would be to establish a primary tumor, allow it to disseminate, surgically remove it, and then simultaneously initiate an adjuvant therapy and randomize the animals to exercise or no exercise prior to the development of tumors (22). The most clinically relevant outcome would be the recurrence of overt disease (22).

A substantial number of observational studies have examined the association between exercise and cancer outcomes in postsurgical patients with early stage cancer (5); however, these studies have rarely distinguished between exercise performed during or after adjuvant therapy. Moreover, they have rarely distinguished between the specific type of adjuvant therapy or the number and sequence of adjuvant therapies. That is, these studies have examined exercise as a cancer treatment in early-stage patients whose micrometastases may be treatment naïve, actively being treated with various treatment modalities, and/or previously treated with various treatment modalities. As noted earlier, current and previous treatments may alter the biology, genetics, and location of remaining cancer and, therefore, modify the effects of exercise as a cancer treatment.

In general, these studies report that higher “postdiagnosis” exercise is associated with lower risks of recurrence, cancer death, and all-cause mortality in breast, prostate, colorectal, and possibly other cancers (5). Moving forward, such observational studies may be designed and analyzed to examine the associations separately between exercise during and after specific types of adjuvant therapies. One advantage of using clinical drug trials for observational exercise research is that the adjuvant therapy is usually restricted to a specific protocol. Such a study allows an answer to a much more clinically relevant question of whether exercise during and/or after a specific adjuvant therapy may lower the risk of recurrence and death. Nevertheless, nested observational exercise studies within randomized drug trials still have substantial limitations and are unlikely to change clinical practice. Properly designed and adequately powered primary randomized exercise trials will be needed.

Many clinical trials have examined the effects of exercise during adjuvant therapy (23–25), however, few have reported on cancer outcomes (10, 24, 25). As one example, the Supervised Trial of Aerobic versus Resistance Training (START) (11) randomized 242 early-stage breast cancer patients initiating chemotherapy to usual care (n=82), aerobic exercise (n=78) or resistance exercise (n=82). In an exploratory analysis, disease-free survival (DFS) (26) after a median follow-up of 89 months was 82.7% for the two exercise groups combined compared with 75.6% for the usual care group, which was not statistically significant (Hazard ratio =0.68, 95% CI=0.37-1.24; log-rank p=0.21). Nevertheless, these data suggest the possibility that adding exercise treatment to adjuvant chemotherapy (i.e., “adjuvant chemo-exercise therapy”) may improve breast cancer outcomes. Although the START trial was not designed or powered to examine cancer outcomes, it included some key features that may inform future trials including the randomized design, prospective exercise interventions, clinically relevant outcomes, and longer-term follow-up.

## Scenario #3: Previously treated micrometastases

The clinical scenario of “previously treated micrometastases” typically occurs after complete surgical removal of an early-stage primary tumor and completion of some regional or systemic adjuvant therapy (e.g., radiotherapy, chemotherapy, immunotherapy, hormone therapy) to treat known or suspected micrometastases. Patients are usually then placed on surveillance. In this scenario where micrometastases have previously been treated, exercise is being proposed as a sequential adjuvant therapy or a “switch” maintenance therapy (i.e., “adjuvant chemotherapy followed by maintenance exercise therapy”). There are many real-world clinical settings where this clinical scenario occurs such as after adjuvant therapy for early-stage breast and colon cancers. The clinical concern in these settings is that the adjuvant therapy may not have eliminated all the remaining cancer cells. The goal of exercise as a cancer treatment in these settings, therefore, would be to eliminate any remaining cancer cells that were not eliminated by the previous adjuvant therapies, possibly through mechanisms previously discussed. This goal poses an additional challenge, however, as any remaining cancer cells were not eliminated by conventional adjuvant therapy and may differ in terms of their biology, location, or newly acquired mutations in response to the previous adjuvant therapies (9). The feasibility of exercise in this clinical scenario is high given the early-stage disease and post adjuvant setting, however, long term exercise adherence may be a challenge. The likelihood of an exercise benefit in this scenario may vary based on real-world clinical settings where the rates of recurrence can vary dramatically. The prospect for an exercise benefit may be higher in settings where the risk of recurrence is higher. Conversely, eliminating cancer cells that were not eliminated by the best currently available adjuvant therapies may be more challenging.

To date, there are very few preclinical exercise studies that have examined exercise as a cancer treatment in this scenario. To examine the effects of exercise on previously treated micrometastases in animal models, the most clinically relevant model would be to establish a primary tumor, allow it to disseminate, surgically remove it, treat the micrometastases with an existing adjuvant therapy, and then randomize the animals without extant tumors to exercise or no exercise (22). The most clinically relevant outcome would be the recurrence of overt disease (22). Exercising animals after chemotherapy treatment has been shown to be feasible, however, these studies have typically focused on chemotherapy toxicities and, therefore, used non-tumor-bearing animals (27).

Most human research on cancer outcomes in these clinical settings has used observational designs (5). As noted earlier, however, most of these studies have not distinguished between exercise performed after surgical resection alone, or during or after adjuvant therapy. Moreover, these studies have typically included survivors who received different types and numbers of adjuvant therapies. Consequently, these studies are restricted to concluding that “postdiagnosis” exercise in early-stage cancer patients are associated with a lower risk of cancer recurrence or death but are unable to provide any more clinically relevant information based on treatment status, the treatment modalities, or the timing of the exercise.

Many clinical trials have examined the effects of exercise after adjuvant therapy (28), however, few have reported on cancer outcomes (10). A novel pilot study (19) examined the effects of exercise on circulating tumor cells in 23 stage I-III colon cancer patients who had completed surgical resection and (mostly) adjuvant chemotherapy in the past 3 years. Results showed that six months of moderate-intensity aerobic exercise at doses of 150 and 300 minutes/week resulted in significant reductions in circulating tumor cells from baseline in the intervention groups, however, the study was not designed to examine clinical outcomes. The Colon Health and Life-Long Exercise Change (CHALLENGE) trial is an ongoing randomized trial in this clinical scenario examining the effects of a 3-year structured exercise program compared to health education materials on disease-free survival among 962 patients with high risk stage II or stage III colon cancer who have completed surgical resection and adjuvant chemotherapy within the past 2-6 months (12). CHALLENGE is designed to answer the specific question of whether exercise as a switch maintenance therapy can lower the risk of recurrence and death in colon cancer patients after surgical resection and a specific adjuvant therapy (i.e., adjuvant chemotherapy followed by exercise maintenance therapy).

## Scenario #4: Treatment naive primary tumors

The clinical scenario of “treatment naïve primary tumors” generally occurs because primary treatment is pending or immediate treatment is deemed unnecessary (i.e., active surveillance). In this scenario where the primary tumor is treatment naïve, exercise is being proposed as a primary therapy or induction therapy. There are many real-world clinical settings where patients wait weeks for primary treatments (e.g., endometrial surgery, prostate surgery, colon surgery) and a growing number of real-world clinical settings where patients are placed on active surveillance (e.g., prostate, urethral, intraocular melanoma). The clinical concern in these settings is the growth and spread of an existing untreated primary tumor. The goal of exercise as a cancer treatment in these clinical settings, therefore, would be to reduce, delay, or prevent the growth and spread of the primary tumor. Exercise may directly affect an existing primary tumor through various systemic and intratumoral mechanisms including immune response, tumor metabolism and physiology, angiogenesis, apoptosis, and DNA synthesis and repair (3). Given the relatively short window between diagnosis and a planned primary treatment, the feasibility of exercise in patients awaiting primary treatment is limited and the prospect for an exercise benefit seems low. Conversely, given the potentially extended time frame in the active surveillance setting (months to years), the high likelihood of cancer progression, and the fact that patients are not being treated, the feasibility of exercise and the prospect for an exercise benefit seems much higher.

To date, this clinical scenario is where most of the animal research has been conducted. In the previous systematic review (3), 26 studies reported 33 models testing the effects of exercise on the growth (and spread) of a treatment naïve primary tumor. Most of the primary tumors were established by subcutaneous injection of cells. Overall, exercise slowed tumor growth in the majority of studies, however, it accelerated tumor growth in 9% of the studies. In general, exercise appears to slow the growth and spread of treatment naïve primary tumors in some animal models, however, it rarely shrinks or eliminates tumors (29). Moreover, it is critically important for researchers to acknowledge the possibility that exercise could worsen outcomes in some specific cancer types and, therefore, may be contraindicated in some clinical settings.

Until recently, there were few opportunities to conduct human studies of exercise in clinical settings of “treatment naïve primary tumors”. There is growing interest in delivering exercise interventions to patients awaiting surgery (30), so-called “prehabilitation” or “window of opportunity” studies. Very few of these studies, however, have focused on cancer outcomes (30). One notable exception is the Pre-Operative Health and Body (PreHAB) study (31) which randomized 49 women with newly diagnosed breast cancer to exercise or a mind-body control group while awaiting surgery (mean 29.3 days). The results showed that exercise treatment prior to surgery did not impact proliferation but it did lead to alterations in gene expression in breast tumors, suggesting that exercise may have a direct effect on breast cancer.

Perhaps the greatest opportunity to study exercise in clinical settings of “treatment naïve primary tumors” is the active surveillance setting. Observational studies and clinical trials may be valuable in this scenario. As one example, the Exercise During Active Surveillance for Prostate Cancer (ERASE) Trial randomized 52 men with localized prostate cancer undergoing active surveillance to 12 weeks of supervised high-intensity interval training or usual care (13). Compared to usual care, the exercise group significantly reduced prostate-specific antigen (PSA) level, PSA velocity, and *in vitro* prostate cancer cell growth. A larger exercise trial with a more clinically relevant endpoint is currently ongoing in this setting (14). The active surveillance setting seems ripe for testing exercise as a primary or induction therapy for treatment naïve primary tumors and may provide one of the greatest opportunities to demonstrate a clinical exercise benefit. Although it may be difficult to demonstrate an overall survival benefit in this scenario, exercise may improve disease control and delay or prevent the need for invasive treatments.

## Scenario #5: Actively treated primary tumors

The clinical scenario of “actively treated primary tumors” occurs most often in settings where an unresected primary tumor is treated with non-surgical therapies such as radiation therapy or chemotherapy either as a primary therapy or neoadjuvant therapy. The clinical goal in this scenario is to reduce or eliminate the primary tumor and possible micrometastases. In the neoadjuvant setting, an excellent response to therapy may allow for a less radical and more definitive surgery or even the avoidance of surgery altogether. When the primary tumor is already being treated, exercise is proposed as a concurrent primary therapy or neoadjuvant therapy (e.g., “neoadjuvant chemo-exercise therapy”, “neoadjuvant radio-exercise therapy”). There are a growing number of real-world clinical settings where patients receive weeks to months of nonsurgical therapies either as a primary therapy (e.g., head and neck, cervical, anal) or as a neoadjuvant therapy (e.g., rectal, esophageal, ovarian, pancreatic, bladder). The clinical concern in these settings is that the treatments may not reduce or eliminate the primary tumor and/or possible micrometastases (i.e., an incomplete or poor response). The goal of exercise as a cancer treatment in these settings, therefore, would be to directly reduce/eliminate the tumor/micrometastases (i.e., an additive effect) or to help the existing therapies reduce/eliminate the tumor/micrometastases (i.e., an interactive effect). Exercise may have direct effects on the tumor as noted earlier, however, it may also enhance treatment efficacy by improving tumor vasculature and perfusion, which may aid in drug delivery to the tumor, or reducing tumor hypoxia, which may improve radiation therapy (32). Exercise should be feasible in this scenario because of the short window for intervention (weeks to months), although treatment side effects may be challenging. The lower likelihood of a cure or complete response in these settings, coupled with the possibility of potentiating the effects of an existing therapy, makes the prospect for an exercise benefit promising.

The clinical scenario of “actively treated primary tumors” has received some attention from preclinical exercise research (4, 33). In general, animal studies have shown that exercise enhances the effects of chemotherapy on tumor growth in an additive, sensitizing, or synergistic manner (4). Further animal research is needed in this increasingly clinically relevant scenario including studies with radiation therapy, hormone therapies, and immunotherapies (33).

In terms of human studies, very few studies have examined exercise during primary or neoadjuvant nonsurgical therapies (34) and even fewer have reported on cancer outcomes. In the Exercise During and After Rectal Cancer Treatment (EXERT) trial (15), 36 rectal cancer patients scheduled to receive neoadjuvant chemoradiotherapy (NACRT) were randomized to usual care (n=18) or exercise (n=18) during and after NACRT (about 12 weeks). Despite limited power and an exploratory analysis, the number of patients achieving a pathologic complete or near complete response was significantly (*p*=0.020) higher in the exercise group (10/18 = 56%) compared to the usual care group (3/17 = 18%). In the Prehabilitation of patients with oEsophageal Malignancy undergoing Peri-operative Treatment (Pre-EMPT) non-randomized trial (35), a structured exercise intervention was compared to a nonexercise group in 39 esophageal cancer patients during neoadjuvant chemotherapy. Compared to the nonexercise group, the exercise group experienced higher rates of tumor regression (75% vs. 37%; p=0.025) and combined tumor and node downstaging (43% vs. 16%; p=0.089). The “actively treated primary tumors” setting seems ripe for more research and may provide another promising opportunity to demonstrate an exercise benefit.

## Scenario #6: Previously treated primary tumors

The clinical scenario of “previously treated primary tumors” may arise if the primary or neoadjuvant nonsurgical therapy did not produce a complete response (**Figure 1B**) and there is no additional immediate treatment or there was an incomplete surgery (debulking). If patients have achieved a complete response, then the scenario reverts back to other management options (**Figure 1**) and exercise may be proposed as a consolidation therapy. In this scenario where the primary tumor remains but has already been treated, exercise is proposed as a second-line or salvage therapy. There are very few real-world clinical settings where patients without a complete response to a primary or neoadjuvant nonsurgical therapy would not receive surgery or further therapy. The clinical concern in these settings is the regrowth and spread of the previously treated primary tumor. The goal of exercise as a cancer treatment in these clinical settings, therefore, would be to slow or reverse tumor growth and spread through mechanisms previously discussed. The added challenge in this scenario, however, is that the primary tumor has not responded well to previous treatments. Given the very short window between an incomplete response and additional treatment, the feasibility of exercise in this scenario and the prospect for an exercise benefit seems low.

To date, there are very few preclinical exercise studies that have examined exercise as a cancer treatment in this clinical scenario, probably because of limited clinical relevance. One study examined the effects of exercise during and after doxorubicin in mice inoculated with lung carcinoma cells and found that exercise reduced the resumption of tumor growth after the cessation of chemotherapy (36). In animal models, a primary tumor would need to be established and then treated with a nonsurgical therapy. Animals with remaining primary tumor would then be randomized to exercise or no exercise and followed for growth and spread of the previously treated primary tumor. Although this clinical scenario may occur infrequently in real-world settings, it may answer a clinically relevant biological question in exercise oncology: “can exercise slow the growth of previously treated primary tumors”? The answer to this question may have implications for whether exercise may be effective only as a first-line treatment or whether it may also be effective after previous cancer treatments have failed (i.e., as a second- or later line therapy). Current opportunities for human research in this clinical scenario, however, seem limited.

## Scenario #7: Treatment naïve metastatic disease

The clinical scenario of “treatment naïve metastatic disease” typically occurs when patients present with *de novo* metastatic disease (including hematological cancers) and there are no viable treatment options available or, more rarely, no immediate treatments are offered. The clinical goal in these patients, therefore, is typically palliative rather than prolonging life. In this scenario where metastatic disease is treatment naïve, exercise is proposed as an induction therapy. Potential mechanisms for an exercise benefit would include the systemic and intratumoral factors mentioned previously, however, the mechanisms would need to address the distinct genetic and epigenetic differences between metastases and primary tumors. There are a number of real-world clinical settings where patients with *de novo* metastatic disease may not receive life-prolonging treatments such as liver, lung, and brain cancers; and fewer settings where they may be placed on observation (e.g., low-grade lymphoma). In settings where no treatments are available, the goal of exercise would also likely be palliative. Exercise may be difficult in these settings due to high symptom burden and a limited life expectancy. The prospects of a clinical benefit seem unlikely.

To date, there are very few preclinical exercise studies that have examined exercise as a cancer treatment in this clinical scenario. As noted previously, some animal studies using metastatic models address both the prevention (occurrence) and treatment (growth) of metastatic tumors with mixed results being reported. To examine the effects of exercise on treatment naïve metastatic tumors in animal models, the most clinically relevant model would be to establish a primary tumor, allow it to disseminate and form metastatic tumors, leave the primary tumor intact (as in a *de novo* model), and then randomize the animals to exercise or no exercise. The most clinically relevant outcomes would be the growth of existing metastatic tumors and the emergence of new metastatic tumors.

Few human studies have examined exercise in patients with treatment naïve metastatic disease. Generally speaking, exercise studies in patients with metastatic disease have not distinguished among patients who were treatment naïve, actively treated, or previously treated (37). Moreover, most human exercise research in these clinical settings has used case studies, qualitative studies, surveys, and feasibility designs, however, few have reported on cancer outcomes (37). The focus has been on palliative outcomes and the research has shown that exercise is feasible for some patients and may provide modest benefits (37).

## Scenario #8: Actively treated metastatic disease

The clinical scenario of “actively treated metastatic disease” occurs where patients with *de novo* or recurrent metastatic disease are treated with non-surgical therapies such chemotherapy or immunotherapy (**Figure 1C**). Patients with metastatic disease rarely receive surgery although selective debulking does occur in some settings. Even in these settings, however, tumors are usually left behind. The clinical goal in most metastatic treatment settings is to prolong survival by reducing the burden of the disease. In some settings, complete remission or cure may be possible (e.g., testicular, lymphoma). In this scenario where the metastatic disease is currently being treated, exercise is proposed as a concurrent therapy. There are many real-world clinical settings where patients with metastatic disease receive nonsurgical cancer treatments including hematologic, testicular, breast, and prostate cancers. The clinical concern in these settings is that the metastatic disease may not respond to treatment (**Figure 1C**). The goal of exercise as a concurrent cancer treatment in these settings, therefore, would be to directly reduce the burden of disease (i.e., an additive effect) or potentiate the effects of existing cancer therapies (i.e., an interactive effect). As noted earlier, exercise may directly affect existing tumors through various systemic and intratumoral mechanisms and may also enhance drug delivery to the tumors *via* improved tumor vasculature and perfusion. The feasibility of exercise in this setting may be challenging given the combination of high symptom burden and treatment side effects (38). Although the window for exercise interventions may be fairly short (weeks to months), the poor outcomes in most of these patient groups and the possibility of potentiating the effects of existing cancer therapies makes the prospect for an exercise benefit at least plausible.

To date, there are very few preclinical exercise studies focused on exercise as a cancer treatment in this clinical scenario. To examine the effects of exercise on actively treated metastatic disease in animal models, the most clinically relevant model would be to establish a primary tumor, allow it to disseminate and form metastatic tumors, leave the primary tumor intact (if a *de novo* model) or remove the primary tumor (if a recurrence model), and then initiate a nonsurgical therapy at the same time as randomizing the animals to exercise or no exercise. The most clinically relevant outcomes would be the growth of existing metastatic tumors and the emergence of new metastatic tumors.

Limited human studies have examined the effects of exercise during treatment for metastatic disease, although some have reported cancer outcomes (37, 39). In a systematic review and meta-analysis of 11 studies of exercise and survival outcomes in patients with advanced cancer, exercise was associated with improved survival in 7 observational studies but not in 4 randomized controlled trials (39). A large observational study (40) examined the associations of physical activity assessed during chemotherapy with survival and progression in 1,218 patients with metastatic colorectal cancer receiving systemic therapy as part of a phase III trial. Compared with patients engaged in less than 3 metabolic equivalent task (MET) hours per week of physical activity, patients engaged in 18 or more MET hours per week experienced a 15% lower risk of death (95% CI, 0.71 to 1.02; p for trend = .06) and a 17% lower risk of progression (95% CI, 0.70 to 0.99; p for trend = .01). This study is an excellent example of using an existing drug trial to answer a more specific question about exercise as a cancer treatment: “is exercise during a specific chemotherapy protocol associated with progression and/or survival in patients with metastatic colorectal cancer”?

The Healthy Exercise for Lymphoma Patients (HELP) trial (16) randomized 122 lymphoma patients to usual care or 12 weeks of supervised aerobic exercise. At the time of the exercise intervention, 54 lymphoma patients were receiving chemotherapy for existing disease. Although not powered to examine treatment response, an exploratory analysis of the patient’s receiving chemotherapy showed that the exercise group had a non-significantly (p=0.24) higher clinical complete response to chemotherapy (13/28 = 46.4%) compared to the usual care group (8/26 = 30.8%). These data suggest the possibility that exercise during chemotherapy may improve treatment response in patients with actively treated metastatic disease. In another trial of 112 patients with advanced lung cancer mostly receiving anti-cancer treatments (78%), it was reported that 8 weekly exercise sessions plus a behavior change program did not affect survival (17).

The Diet Restriction and Exercise-induced Adaptations in Metastatic Breast Cancer (DREAM) (41) is an ongoing trial examining the effects of a combined diet and exercise intervention performed during intravenous chemotherapy on tumor burden in 50 breast cancer patients with measurable metastases. A novel aspect of this study is that the exercise intervention is being performed during the chemotherapy infusions. Another ongoing trial in this clinical oncology scenario is the Intense Exercise for Survival among Men with Metastatic Prostate Cancer (INTERVAL-GAP4) trial (18) which is examining the effects of a 2-year exercise program on overall survival in 866 men with metastatic prostate cancer who have been previously treated and/or are being actively treated with hormone therapy and/or chemotherapy. This trial is the first prospective randomized trial designed to examine exercise and survival in men with metastatic prostate cancer.

## Scenario #9:Previously treated metastatic disease

The clinical scenario of “previously treated metastatic disease” typically occurs if patients have not achieved a complete remission after treatments (**Figure 1C**) and, therefore, previously treated metastatic disease remains. If patients have achieved a complete remission, the scenario reverts back to surveillance with a focus on micrometastases (**Figure 1C**) and exercise may be proposed as a consolidation therapy. The clinical goal in patients without a complete remission is to further delay the growth and spread of the previously treated disease. In this scenario, exercise treatment is proposed as a later-line therapy or salvage therapy. Exercise may directly affect existing tumors through various systemic and intratumoral mechanisms, however, the added challenge is that the metastatic tumors have not responded well to previous treatment which may have altered the biology, genetics, and location of remaining tumors. There are many real-world clinical settings where patients with metastatic disease do not achieve a complete remission such as metastatic breast, colon, pancreas, and lung cancers. The goal of exercise in these settings may be palliative or focused on prolonging life. Exercise may be difficult in these settings due to high symptom burden, lingering treatment side effects, and a limited life expectancy. The likelihood that exercise could provide a survival benefit may depend on whether patients are still responding to treatments even without a complete remission.

To date, there are very few preclinical exercise studies focused on exercise as a cancer treatment in this clinical scenario. To examine the effects of exercise on previously treated metastatic disease in animal models, the most clinically relevant model would be to establish a primary tumor, allow it to disseminate and form metastatic tumors, leave the primary tumor intact (if a *de novo* model) or remove the primary tumor (if a recurrence model), complete a nonsurgical therapy, and then randomize the animals to exercise or no exercise. The most clinically relevant outcomes would be the growth of existing metastatic tumors and the emergence of new metastatic tumors.

As noted earlier, human exercise studies in patients with metastatic disease have typically included patients with mixed treatment status and focused on feasibility and palliative outcomes (37). Study designs have consisted of case studies, qualitative studies, surveys, and feasibility studies designs. Few conclusions can be drawn from these studies and the role of exercise as a cancer treatment remains unclear. The INTERVAL-GAP4 trial (18) noted earlier includes some patients previously treated for metastatic disease who still have additional lines of therapy available.

## Application of the EXACT framework

The EXACT framework describes nine distinct clinical oncology scenarios that can be applied to any setting consisting of a specific type of cancer and a specific treatment protocol. For example, if a primary tumor of a specific type of cancer is removed surgically and then immediately treated with a specific radiation therapy protocol, the EXACT framework identifies two possible roles of exercise as a cancer treatment in that setting: (a) a concurrent adjuvant therapy to treat micrometastases from a specific type of cancer that is being actively treated by a specific radiation therapy protocol (i.e., concurrent adjuvant radio-exercise therapy) or (b) a sequential adjuvant therapy or a switch maintenance therapy (depending on dosing and scheduling) to treat micrometastases from a specific type of cancer that has been previously treated by a specific radiation therapy protocol (i.e., adjuvant radiation therapy followed by adjuvant or maintenance exercise therapy). Given there are over 200 (sub)types of cancer and a vast number of cancer treatment protocols, there are an infinite number of unique clinical settings in which exercise could be tested as a cancer treatment (**Table 6**). Moreover, new subtypes of cancer and new treatment protocols are introduced on a regular basis. The EXACT framework provides a simple taxonomy for systematically evaluating the potential role of exercise as a cancer treatment across distinct clinical oncology settings that involve specific cancer types and specific treatment protocols.

**Table 6:** Theoretical combinations of disease/tumor status, diseases, treatment status, and treatment modalities.

Tumor Disease Status	X	Diseases	X	Treatment Status^1^	X	Treatment Modalities^2^
						Radiation Therapy
Primary tumor removed				Treatment naïve		Chemotherapy
Primary tumor present	X	>200 types/subtypes	X	Actively treated	X	Hormone therapy
Metastatic disease present				Previously treated		Immunotherapy
						Targeted therapy

^1^Treatment status applies to first-line and later-line therapies. ^2^Each treatment modality has many different types, doses, and schedules that can be combined with other treatment modalities in a concurrent or sequential manner.

## Limitations and conclusions

There are important limitations of both the EXACT framework as well as the research overview in the present paper. First, the EXACT framework may not cover all real-world clinical oncology settings, however, the nine scenarios described here likely cover the vast majority. Second, real world clinical settings within the same clinical scenario will differ markedly based on cancer type (cell line) and treatment protocol. Exercise research will need to address each real-world clinical setting and determine the extent to which research within the same clinical scenario may be generalized across cancer types and/or treatment protocols. Third, the proposed treatment statuses of treatment naïve, actively treated, and previously treated are not mutually exclusive as some patients may be actively treated after being previously treated (i.e., second or third-line therapies), and some patients may be considered treatment naïve for metastatic disease after being treated for local disease. Moreover, the influence of treatment status on exercise effects may vary dramatically depending on the treatment modality and the specific treatment protocol. Again, researchers will need to determine the extent to which research within one treatment modality/protocol can be generalized to other treatment modalities/protocols. Fourth, the EXACT framework is an organizational framework and does not address the biological mechanisms for potential exercise effects in each of the distinct clinical oncology scenarios or specific clinical oncology settings. Finally, exercise may play multiple roles as a cancer treatment within the same clinical scenario. For example, exercise treatment may be administered as concurrent adjuvant therapy and then as a continuous maintenance therapy in the same clinical setting.

In terms of the research overview, this paper did not provide a systematic review of preclinical and human research for each of the nine distinct clinical oncology scenarios. Rather, it summarized previous systematic reviews and highlighted a few select studies. Some systematic reviews and notable studies may have been missed that address these scenarios. The primary purpose of this paper was to propose a conceptual framework as a way of organizing existing research and guiding future research. The research overview attempted to highlight the general state of research within each clinical scenario and provide some examples but was by no means comprehensive. Future systematic reviews of preclinical and/or human research may be organized by the distinct clinical scenarios highlighted in the EXACT framework. Finally, the proposed clinically relevant animal models for some of the clinical scenarios may be technically challenging or entirely unfeasible at this time. Continued development of animal models may allow for more clinically relevant scenarios in which to test exercise as a cancer treatment (22).

In conclusion, developing and testing exercise as a cancer treatment requires an appreciation of current clinical oncology settings for both animal and human research. Given the diversity of cancers and treatments, there are thousands of unique clinical oncology settings in which exercise could be tested for clinical benefit. The EXACT framework provides one strategy for thinking systematically about the role of exercise as a cancer treatment across a diverse range of cancer types and treatment protocols by highlighting tumor/disease status and treatment status. Many current preclinical, observational, and clinical exercise studies are limited because they mix across these clinical scenarios by including animals/patients with different tumor/disease statuses and/or different treatment statuses. Moreover, several of the clinical scenarios have received limited research attention from preclinical and clinical researchers. Future research should focus within a single clinical scenario while addressing a specific cancer type and treatment protocol. The EXACT framework might also prove useful for guiding research on other lifestyle, behavioral, and complementary therapies purported to act as cancer treatments.

## Data availability statement

The original contributions presented in the study are included in the article/Supplementary Material. Further inquiries can be directed to the corresponding author.

## Author contributions

KC conceived the paper and the EXACT Framework and wrote the first draft of the manuscript. CB provided substantive feedback on medical oncology terminology and treatments and critically revised the framework and manuscript. All authors contributed to the article and approved the submitted version.

## Funding

KC is supported by the Canada Research Chairs Program and a Foundation Grant from the Canadian Institutes of Health Research (159927). CB is supported by the Canada Research Chairs Program.

## Conflict of interest

The authors declare that the research was conducted in the absence of any commercial or financial relationships that could be construed as a potential conflict of interest.

## Publisher’s note

All claims expressed in this article are solely those of the authors and do not necessarily represent those of their affiliated organizations, or those of the publisher, the editors and the reviewers. Any product that may be evaluated in this article, or claim that may be made by its manufacturer, is not guaranteed or endorsed by the publisher.
